# Development and validation of risk prediction and neural network models for dilated cardiomyopathy based on WGCNA

**DOI:** 10.3389/fmed.2023.1239056

**Published:** 2023-10-05

**Authors:** Wei Yu, Lingjiao Li, Xingling Tan, Xiaozhu Liu, Chengliang Yin, Junyi Cao

**Affiliations:** ^1^Chongqing Medical University, Chongqing, China; ^2^Faculty of Medicine, Macau University of Science and Technology, Macau, China; ^3^Department of Medical Quality Control, The First People’s Hospital of Zigong City, Zigong, China

**Keywords:** dilated cardiomyopathy, weighted gene co-expression network analysis, risk prediction model, neural network model, bioinformatics

## Abstract

**Background:**

Dilated cardiomyopathy (DCM) is a progressive heart condition characterized by ventricular dilatation and impaired myocardial contractility with a high mortality rate. The molecular characterization of DCM has not been determined yet. Therefore, it is crucial to discover potential biomarkers and therapeutic options for DCM.

**Methods:**

The hub genes for the DCM were screened using Weighted Gene Co-expression Network Analysis (WGCNA) and three different algorithms in Cytoscape. These genes were then validated in a mouse model of doxorubicin (DOX)-induced DCM. Based on the validated hub genes, a prediction model and a neural network model were constructed and validated in a separate dataset. Finally, we assessed the diagnostic efficiency of hub genes and their relationship with immune cells.

**Results:**

A total of eight hub genes were identified. Using RT-qPCR, we validated that the expression levels of five key genes (ASPN, MFAP4, PODN, HTRA1, and FAP) were considerably higher in DCM mice compared to normal mice, and this was consistent with the microarray results. Additionally, the risk prediction and neural network models constructed from these genes showed good accuracy and sensitivity in both the combined and validation datasets. These genes also demonstrated better diagnostic power, with AUC greater than 0.7 in both the combined and validation datasets. Immune cell infiltration analysis revealed differences in the abundance of most immune cells between DCM and normal samples.

**Conclusion:**

The current findings indicate an underlying association between DCM and these key genes, which could serve as potential biomarkers for diagnosing and treating DCM.

## 1. Introduction

Dilated cardiomyopathy (DCM) is a heart muscle disease characterized by ventricular dilatation and impaired myocardial contraction, with an estimated incidence of about 1/2500–1/250 (1, 2). The pathogenic factors of DCM comprise genetic elements, infected or non-infected inflammation, poisoning (including alcohol, etc.), endocrine and metabolic disorders, and psychic trauma (2). DCM can also worsen into severe congestive heart failure, endangering the lives of patients and imposing a tremendous economic and societal burden. However, under the current therapeutic paradigm, the only effective treatment option available to cure DCM is a heart transplant (3, 4). Therefore, identifying key genes associated with DCM progression is essential to preventing a poor prognosis.

Microarray is an effective high-throughput sequencing method used for gene function analysis that combines both high specificity and high sensitivity (5). It can measure the entire spectrum of gene expression levels, allowing for the identification of differentially expressed genes (DEGs) and alterations in biological processes such as in DCM, which can be used for its diagnosis and treatment (6). Furthermore, microarray technology, in combination with extensive bioinformatics analysis, has been widely employed in identifying novel biomarkers and their associations with a wide range of diseases to further the understanding of disease pathophysiology and develop effective therapeutic approaches (7, 8). Weighted gene co-expression network analysis (WGCNA) is a well-established method in bioinformatics applications for identifying underlying mechanisms, potential biomarkers, or therapeutic targets for a variety of disorders (9). Therefore, the analysis of DCM datasets using bioinformatics methods may offer a new perspective on the pathogenesis of DCM.

Here, we used bioinformatics methods to investigate the molecular characterization of DCM. The “sva” software was used to minimize batch differences that were created during the merging process of two datasets (GSE57338, GSE120895) into a single comprehensive dataset. The “LIMMA” package was allowed for the identification of differentially expressed genes. Hub genes were identified and analyzed using WGCNA, enrichment analysis, and protein–protein interaction (PPI) networks. Following validation in an animal model, the hub genes were used to create a prediction and genetic diagnostic models for DCM to assess the diagnostic values of the genes.

## 2. Methods

### 2.1. Pre-processing of DCM dataset

Three DCM datasets (GSE57338, GSE120895, and GSE116250) were obtained from NCBI’s Gene Expression Omnibus^1^ (10). The details of each dataset are shown in Supplementary Table S1. The initial processing of GSE57338 and GSE120895 was carried out in R (version 4.1.1), which included dataset consolidation, background calibration, normalization, and log2 transformation (11). Multiple probes corresponding to the same gene were taken as the gene expression value. The “sva” R package was also used for minimizing batch impacts and other undesired variances (12). Moreover, DEGs for DCM were screened using the “LIMMA” package, with an adjusted value of *p* < 0.01 and |log2 Fold change (FC)| ≥ 0.5 (13). In addition, a separate dataset (GSE116250) was used for subsequent validation analysis.

### 2.2. Identification of the most relevant modules for DCM using WGCNA analysis

A network of gene co-expression was created using WGCNA (9) to examine gene interactions. Genes with less than 25% variation between samples were first eliminated, and the remaining genes were then imported into WGCNA for subsequent analysis. Further, using the pick-Soft-Threshold function, adjacency was generated based on the soft thresholding power β, which was determined by co-expression similarity. The corresponding dissimilarity (1-TOM) was determined after converting the adjacency relationship into a topological overlap matrix (TOM). Hierarchical clustering and the dynamic tree-cut approach were employed to carry out module detection. We used average linkage hierarchical clustering with a minimum size (gene group) of 100 for the gene dendrogram to classify genes with comparable expression profiles into gene modules (14). Additionally, module membership (MM) and gene significance (GS) were calculated for modules associated with clinical attributes. A visualization of the eigenstate network was also performed. The module’s genetic data were used for subsequent analysis. By reviewing the literature, we obtained 172 genes that were of interest to us. These interesting genes (IGs) were subsequently used for the acquisition of common genes (CGs). Thereafter, CGs were identified by intersecting the DEGs screened in the combined dataset, the most important module’s genes, and genes of interest.

### 2.3. GO and KEGG enrichment analysis of common genes

The web-based platform Metascape (version 3.5; http://metascape.org) provides a comprehensive tool for researchers to annotate and analyze gene functions (15). It has been used for enrichment analysis to carry out the Gene Ontology (GO) and the Kyoto Encyclopedia of Genes and Genomes (KEGG) for the CGs. The enriched GO terms included ontologies for biological processes, cellular components, and molecular structures. Here, a value of *p* < 0.05 and count ≥ 2 were set as the cutoff value.

### 2.4. Protein–protein interaction network construction

The PPI interaction network of CGs was created using the STRING database (version 11.5; www.string-db.org), wherein a minimum interaction score of 0.15 was determined as the critical value (16). The PPI network was then visualized using Cytoscape. Three different algorithms of the Cytoscape plug-in, CytoHubba, were applied to obtain the hub genes of the PPI network. Further analysis was conducted using the Maximum Clique Centrality (MCC), Maximum Neighborhood Component (MNC), and Edge Percolated Component (EPC) algorithms. The top eight genes from each algorithm were plotted in a Venn diagram, and overlapping genes between the three groups were considered as the hub genes.

### 2.5. DCM animal model establishment

All animals were provided by the Animal Experimental Center of Chongqing Medical University (Chongqing, China). This study was approved by the Chongqing Medical University Animal Welfare Committee. As shown in previous studies, the DCM model was established by intraperitoneally injecting mice with DOX (17, 18). Male C57BL/6 mice (8 weeks; 22–24 g) were kept on a 12:12 h light–dark cycle in a room at 25°C and 50% relative humidity with free access to food and water. Mice were randomly assigned into two groups: control (*n* = 6) and DOX (*n* = 6). Mice in the DOX group received intraperitoneal administration of DOX hydrochloride (cat. no. 25316–40–9; Macklin; 4 mg/kg) once a week for 8 weeks. The control group received an identical dosage of saline. Successful establishment of the DCM model was confirmed by gradual cardiac functional deterioration and ventricular dilation in mice.

### 2.6. Transthoracic echocardiography

Mice were initially anesthetized with 5% isoflurane (cat.no. 792632; Sigma-Aldrich; Merck KGaA), and the anesthetic effect was sustained at a concentration of 1.5% isoflurane. Thereafter, transthoracic echocardiography (cat. no. VINNO 6LAB) was performed in M-mode with a 23-MHz transducer. Both long-axis and short-axis images were used to evaluate the left ventricle (LV) echocardiography. The end-systolic and end-diastolic dimensions were assessed by the phases corresponding to the ECG T and R waves, respectively (19). M-mode LV internal diameter end systole/diastole (LVIDs/d), LV posterior wall end diastole (LVPWd), and interventricular septal end diastole (IVSd) were measured thrice. Fractional shortening and ejection fractions were calculated, as mentioned previously (19).

### 2.7. RNA extraction and RT-qPCR

Mice were euthanized by general anesthesia isoflurane followed by cervical dislocation. The hearts were quickly extracted by opening the thorax and placed into the cold (4°C) saline. The total RNA was isolated from the heart tissues using the Animal Total RNA Isolation Kit (cat. no.RE-03014; Foregene). For reverse transcription, 5X All-In-One RT MasterMix (cat. G492; Abm.) was used. The cDNA was kept at room temperature for 5 min. The reaction was inactivated at 85°C for 5 min followed by incubation at 25°C for 10 min and at 42°C for 15 min. Also, qPCR was performed using the EvaGreen Express 2 × qPCR MasterMix-No Dye (cat. MasterMix-ES; Abm) and SLAN-96S Real-Rime PCR system (Shanghai Hongshi Medical Technology Co., Ltd.). The primers for GAPDH (internal control), latent transforming growth factor beta binding protein 2 (LTBP2), asporin (ASPN), immunoglobulin superfamily contain leucine-rich repeat (ISLR), microfibril associated protein 4 (MFAP4), fibronectin type III domain containing 1 (FNDC1), podocan (PODN), HtrA serine peptidase 1 (HTRA1), and fibroblast activation protein (FAP) were synthesized by Sangon Biotech (Shanghai) Co., Ltd. (Table 1). The 2^–ΔΔCT^ method was used to standardize all samples to GAPDH levels, and the process was repeated three times for each sample (20).

**Table 1:** Primer sequences.

Gene	Forward (5′→ 3′)	Reverse (5′→ 3′)
GAPDH	GGTTGTCTCCTGCGACTTCA	TGGTCCAGGGTTTCTTACTCC
LTBP2	GCTCACCGGGAGAAATGTCTG	CAGGTTTGATACAGTGGTTGGT
ASPN	AAGGAGTATGTGATGCTACTGCT	ACATTGGCACCCAAATGGACA
ISLR	CTGTGCCTATCGTGACCTAGA	CCACCGAGCGGATCTCATT
MFAP4	GGCGTGTATCTCATCTACCCC	TCACTGAGCCGTTGAATCTTTT
FNDC1	GGTGGAGTATTACAACATTGCCT	AAGGAGTGTGTCTCCGCATTC
PODN	GCATTTGAGCATCTTACTAGCCT	CACAGACCTCAGATTTGGCTTT
HTRA1	TAGCGACGCCAAGACCTACA	TGACGCAAACTGTTGGGATCT
FAP	GTCACCTGATCGGCAATTTGT	TCGTAGATGTAGTATGTCGCTGT

### 2.8. Construction of risk prediction model and artificial neural network model

We performed a multivariate logistic regression analysis based on the experimentally validated key genes and constructed a risk prediction model using the “rms” package (21). A nomogram was also developed to predict the risk score of each individual. Additionally, the sensitivity and specificity of the model were assessed using ROC and calibration curve analysis. Similarly, an artificial neural network model of five validated key genes was constructed using the R software package “neuralnet” (22). A classification model for DCM was established utilizing data on gene weights and four implicit layers as model parameters. The disease classification score in this model was calculated by adding the weight scores multiplied by the relevant genes’ expression levels. The ROC curve was then plotted to assess the sensitivity and accuracy of the model. A different dataset (GSE116250) taken as a test group was used for the validation of the two models mentioned above, and their respective ROC curves were plotted separately.

### 2.9. Evaluation of diagnostic efficiency of hub genes

To better understand the diagnostic efficiency of nomogram constitutive genes for DCM, the expression of each gene was compared between the control and experimental groups using the Wilcoxon sum-rank test, and their corresponding ROC curves were plotted. Additionally, these genes were verified in the validation dataset (GSE116250).

### 2.10. Evaluation of immune cell infiltration in patients with dilated cardiomyopathy

We evaluated the relative abundance of immune cells infiltrating between samples from DCM patients and healthy individuals using the ssGSEA approach to determine the composition of immune cells in the cardiac immune microenvironment. In addition, a correlation test between immune cells was conducted using the “corrplot” R package and a heatmap depicting immune cell correlations was plotted (23). The correlation between different immune cell types was analyzed by Spearman’s method and illustrated as a heatmap. The Wilcoxon test was used to examine the immune cell differences between samples from DCM patients and those from healthy individuals. The results were then presented using a violin diagram. Additionally, the correlation between five validated hub genes and immune cells was calculated using Spearman’s method. Based on the results obtained from gene and immune cell correlation, the lollipop diagram was plotted to highlight the gene with the highest correlation to immune cells.

### 2.11. Statistical analysis

GraphPad Prism (version 9.3.0) was used for statistical analysis. Data are presented as the mean ± standard deviation (SD). The two groups were compared using an independent sample *t*-test. In addition, the Wilcoxon sum-rank test was performed for the boxplot of the average difference in the expression of hub genes. The relationship between immune cells and between immune cells and hub genes was examined using Spearman’s method. The Wilcoxon test was performed to identify immune cell differences between samples from healthy individuals and DCM patients. A value of *p* < 0.05 was considered statistically significant.

## 3. Results

### 3.1. Determination of the most relevant modules and common genes

**Figure 1:**
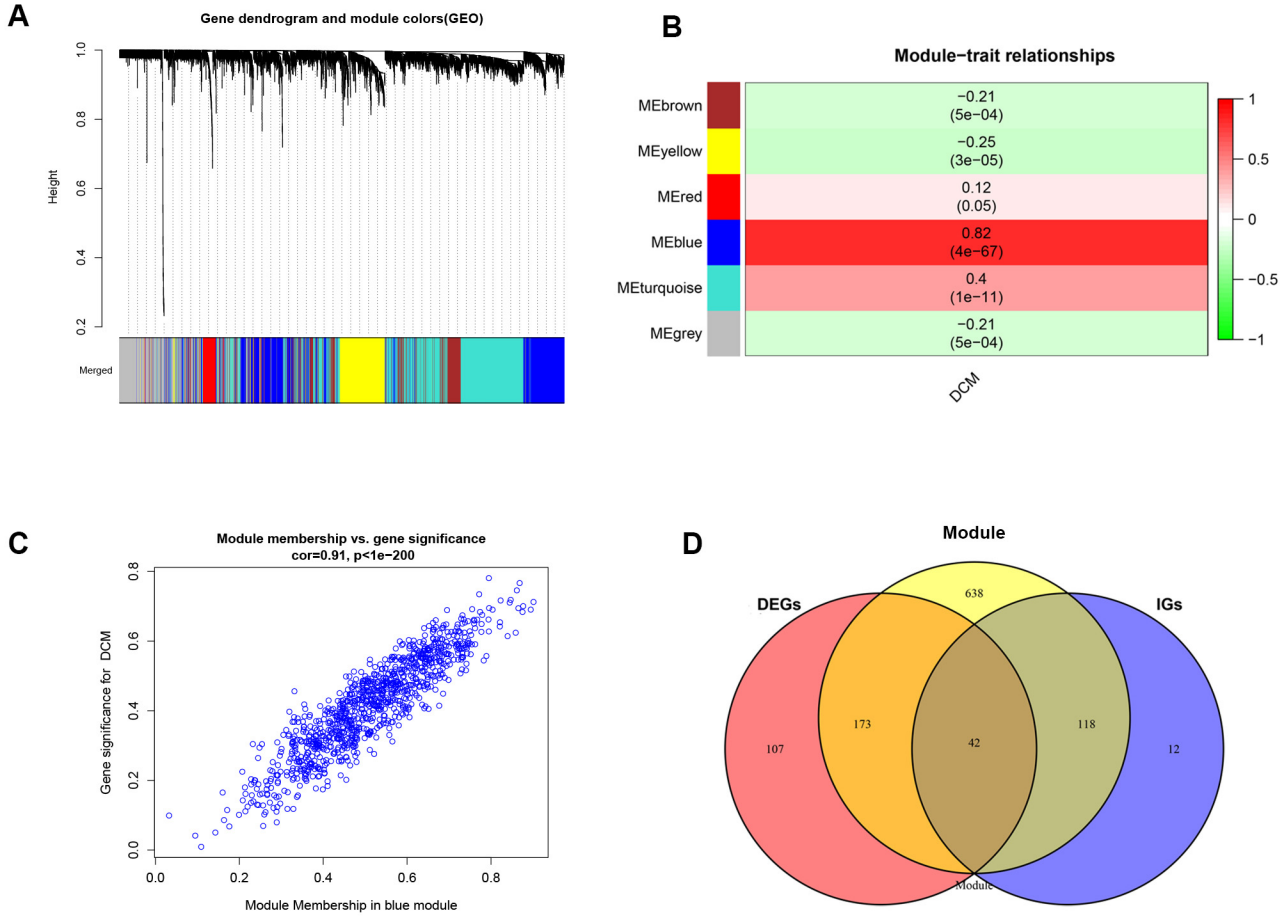
Implementation of WGCNA analysis and acquisition of common genes. **(A)** The gene co-expression module under the gene tree is represented using different colors. **(B)** Heatmap depicts the association between modules and DCM. The numbers inside and outside of the brackets represent *p* values and correlation coefficients, respectively. **(C)** A correlation map between the gene significance and module membership in the blue module. **(D)** Venn diagram of common genes acquisition. DEGs, differentially expressed genes; DCM, dilated cardiomyopathy; WGCNA, Weighted Gene co-expression network analysis; IGs, interesting genes.

We analyzed the differential expression of genes in the combined dataset using the “LIMMA” program package to better understand the changes at the gene level in DCM. The merged dataset identified 322 DEGs, comprising 164 upregulated and 158 downregulated genes. The heatmap and volcano plot of DEGs are shown in Supplementary Figures S1A,B, respectively. Genes exhibiting variance greater than 25% in the merged dataset were imported to the WGCNA. The “pickSoftThreshold” function from the “WGCNA” package was used to calculate the power parameter in the range of 1–20. To ensure the reliability of the scale-free network β = 7 (scale-free *R*^2^ = 0.85) was considered as a soft threshold in our study (Supplementary Figure S2B). Additionally, the threshold was set to 0.25, and the green module was merged into the blue module (Supplementary Figure S2A). Figure 1A illustrates a total of six modules, each comprising genes with comparable co-expression characteristics. A random selection of colors was made to distinguish the different modules. The signature genes from the blue module (*r* = 0.82; *p* = 4 × 10^−67^) showed the highest positive correlation and significant association with DCM when compared to other modules (Figure 1B). As a result, the blue module containing 971 genes was identified as being crucial for DCM and hence utilized for subsequent analyses. Moreover, correlations between gene module memberships (MMs) and gene significances (GSs) were evaluated in the blue module. In the blue module, there were significant positive correlations (*r* = 0.91, *p* < 1 × 10^−200^) between the MM and GS of genes (Figure 1C). CGs were obtained by utilizing the intersection of the DEGs screened by the LIMMA package, the genes in the blue module, and the genes in IGs (Figure 1D).

### 3.2. Enrichment analyses of the common genes

**Figure 2:**
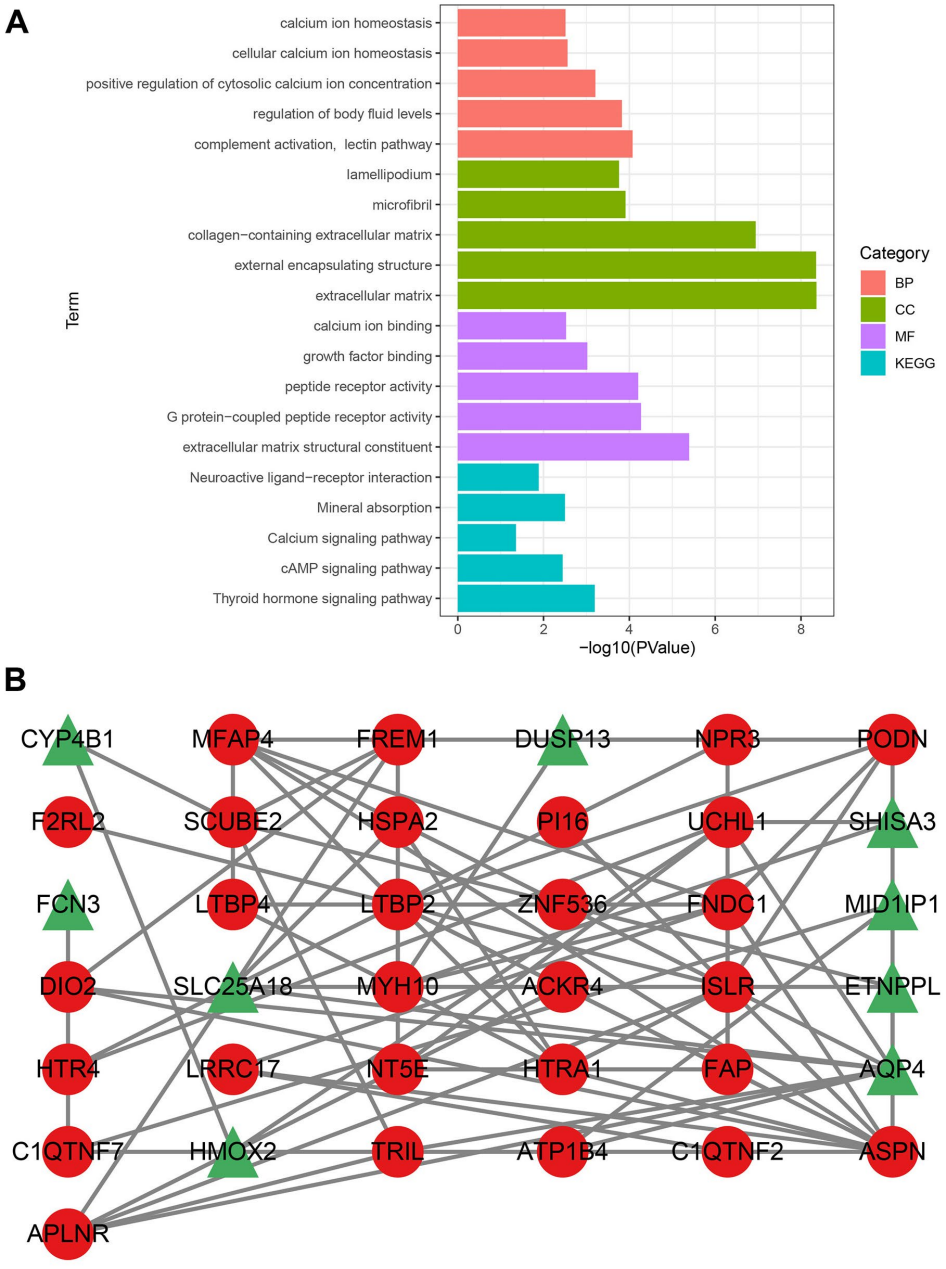
Analysis of gene enrichment pathways and construction of PPI networks. **(A)** Gene enrichment analysis of CGs. The color green denotes CC, the color red denotes BP, the color purple denotes MF, and the color blue denotes the KEGG pathway. **(B)** Upregulated genes are indicated by nodes with red circles, whereas downregulated genes are indicated by nodes with green triangles. CGs, common genes; CC, cellular component; BP, biological process; MF, molecular function; GO, gene ontology; and KEGG, Kyoto encyclopedia of genes and genomes.

Functional enrichment analysis revealed that the CGs were primarily implicated in calcium-related pathways, such as calcium ion homeostasis, cellular calcium ion homeostasis, positive regulation of cytosolic calcium ion concentration, calcium ion binding, and calcium signaling pathway (Figure 2A). Calcium is essential for the normal physiological function of the heart. These findings suggest that calcium-related pathways may affect the onset and progression of DCM.

### 3.3. PPI network

The PPI network obtained by STRING was visualized using Cytoscape. Following the removal of the isolated nodes, a PPI network with 37 nodes and 74 edges, containing 28 upregulated and nine downregulated genes was established (Figure 2B). Three CytoHubba plug-in algorithms (MCC, MNC, and EPC) were then applied to screen the hub genes of the PPI network. The top eight genes selected from each algorithm were used for Venn diagram analysis (Supplementary Figure S3). Thereafter, LTBP2, ASPN, ISLR, MFAP4, FNDC1, PODN, HTRA1, and FAP were identified as hub genes. Our study indicates that these hub genes might function as potential biomarkers, leading to the development of novel therapeutic options for the diseases being studied.

### 3.4. Validation of the hub genes in a DCM mouse model

**Figure 3:**
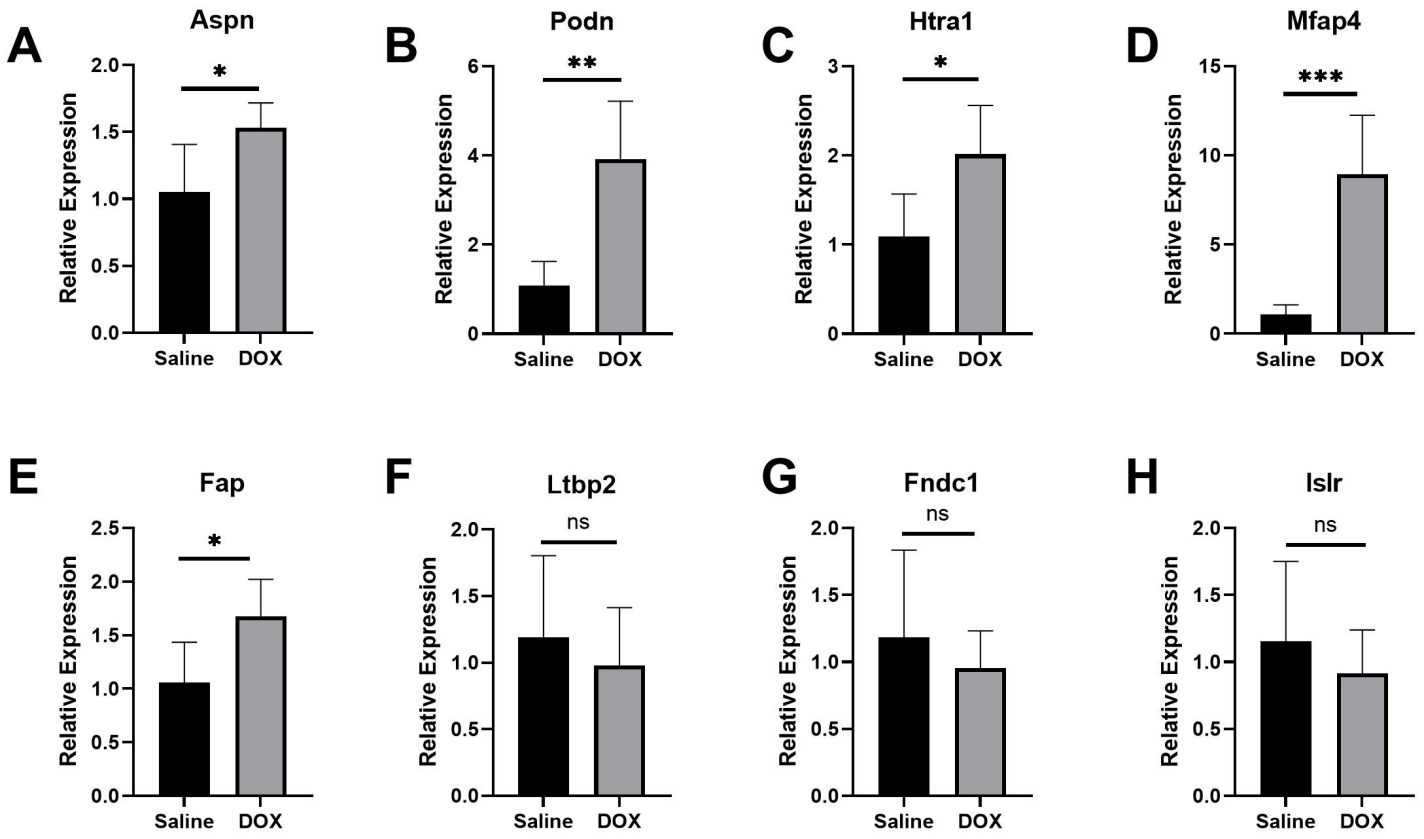
Validation of the hub genes in the DCM mice model. **(A–H)** Relative expression of Aspn, Podn, Htra1, Mfap4, Fap, Ltbp2, Fndc1, and Islr in DCM mice. DCM group, *n* = 5; control group, *n* = 5. Data are presented as the mean ± standard deviation. DOX, doxorubicin (^***^represents *p* < 0.001; ^**^represents *p* < 0.01; ^*^represents *p* < 0.05; and ns represents *p* > 0.05).

An *in vivo* model of DCM was created by repeatedly injecting mice with DOX. As shown in previous studies (24, 25), the successful establishment of the DCM model was marked by exacerbated cardiac dysfunction and ventricular dilatation in mice. Mice administered with cumulative doses of 20 mg/kg DOX exhibited impaired heart function (Supplementary Figures S4A,B; *p* < 0.001), dilated left ventricles (Supplementary Figures S4C,D; *p* < 0.001), and thin ventricular walls (Supplementary Figures S4E,F; *p* < 0.001), similar to the pathophysiological symptoms of human DCM (26). Using RT-qPCR, the levels of mRNA expression of the following genes were assessed in mice hearts: LTBP2, ASPN, ISLR, MFAP4, FNDC1, PODN, HTRA1, and FAP (DCM group, *n* = 5; control group, *n* = 5). Unfortunately, a sample from the DCM group had to be discarded due to a processing error. The current findings reveal that the levels of mRNA expression of hub genes (ASPN, MFAP4, PODN, HTRA1, and FAP) were significantly higher in the hearts of DOX treated mice as compared to controls (Figures 3A–E; *p* < 0.05), whereas there were no significant differences in the expression levels of the remaining genes (LTBP2, FNDC1, and ISLR; Figures 3F–H; *p* > 0.05). The *in vivo* changes in the expression of these hub genes provided additional support for the microarray findings, suggesting a possible link between these genes and DCM.

### 3.5. Construction of risk prediction model and artificial neural network based on hub genes

**Figure 4:**
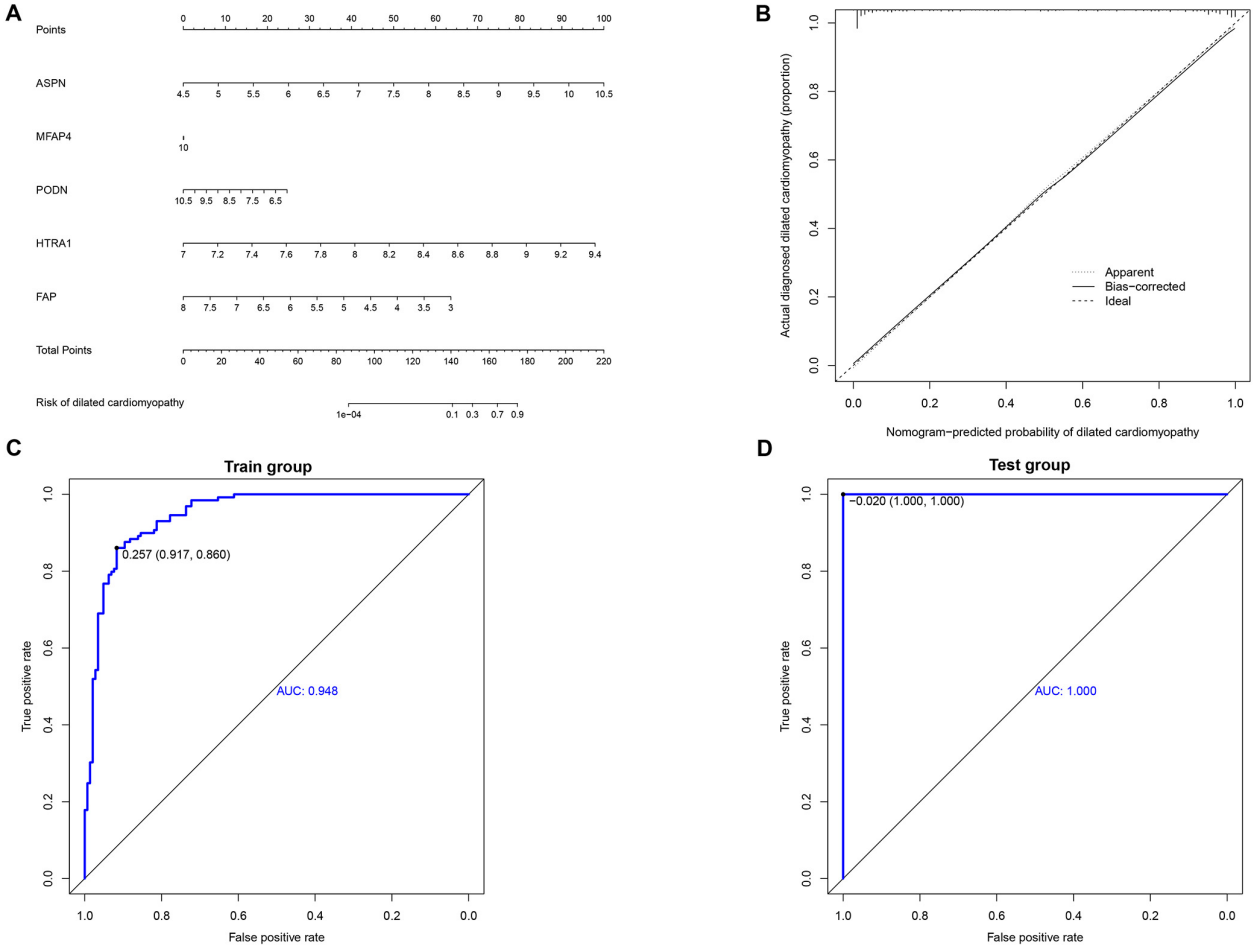
Construction and validation of nomogram. **(A)** Development of a prediction nomogram based on validated hub genes. **(B)** Calibration curves for predicting the risk of dilated cardiomyopathy nomogram. **(C)** ROC curve analysis of risk prediction model in train group. **(D)** ROC curve analysis of risk prediction model in the test group.

**Figure 5:**
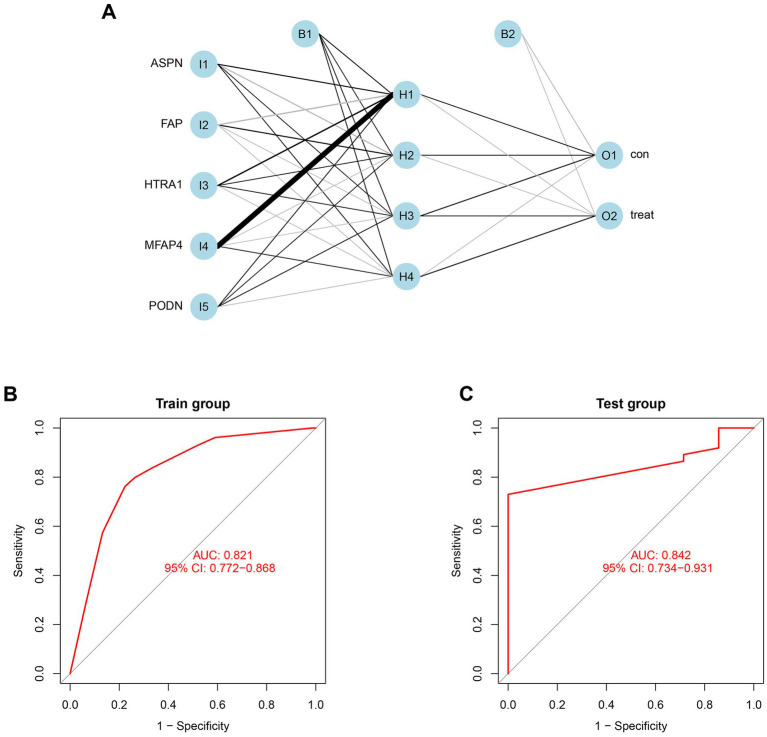
Construction and validation of neural network model. **(A)** Result from neural network visualization. **(B)** ROC curve of the neural network model in train group (GSE57338). **(C)** ROC curve of the neural network model in the test group (GSE116250).

Using the independent predictors mentioned above, a risk prediction model was developed and presented as a nomogram (Figure 4A). The calibration curve for the DCM risk nomogram, which is used to predict DCM risk, was shown to have a high degree of agreement in this group (Figure 4B). The ROC curve of the risk prediction model depicted an area under the curve (AUC) of 0.948 (Figure 4C), suggesting a high accuracy and sensitivity of the model. These genes were then used to build a neural network model, with the scores and weights of the feature genes comprising the input layer of this model. The extracted feature information from the input layer was used to create the implicit layer. The output layer determined if the samples belonged to either the normal or DCM group (Figure 5A). Results of the model in the training group showed that the control group had 112 correct predictions out of 144 samples (77.8% accuracy), while the DCM group had 98 accurate predictions out of 129 samples (76.0% accuracy). The AUC value of the ROC curve was calculated to be 0.821 (Figure 5B). To validate the prediction accuracy of these two models, additional models were built and ROC curves were plotted in the test group (GSE116250) using the same methods. The AUC values calculated from the ROC curve plotted by the risk prediction model (Figure 4D) and the neural network model (Figure 5C) were 1 and 0.842, respectively.

### 3.6. Verification and efficacy assessment of hub genes

**Figure 6:**
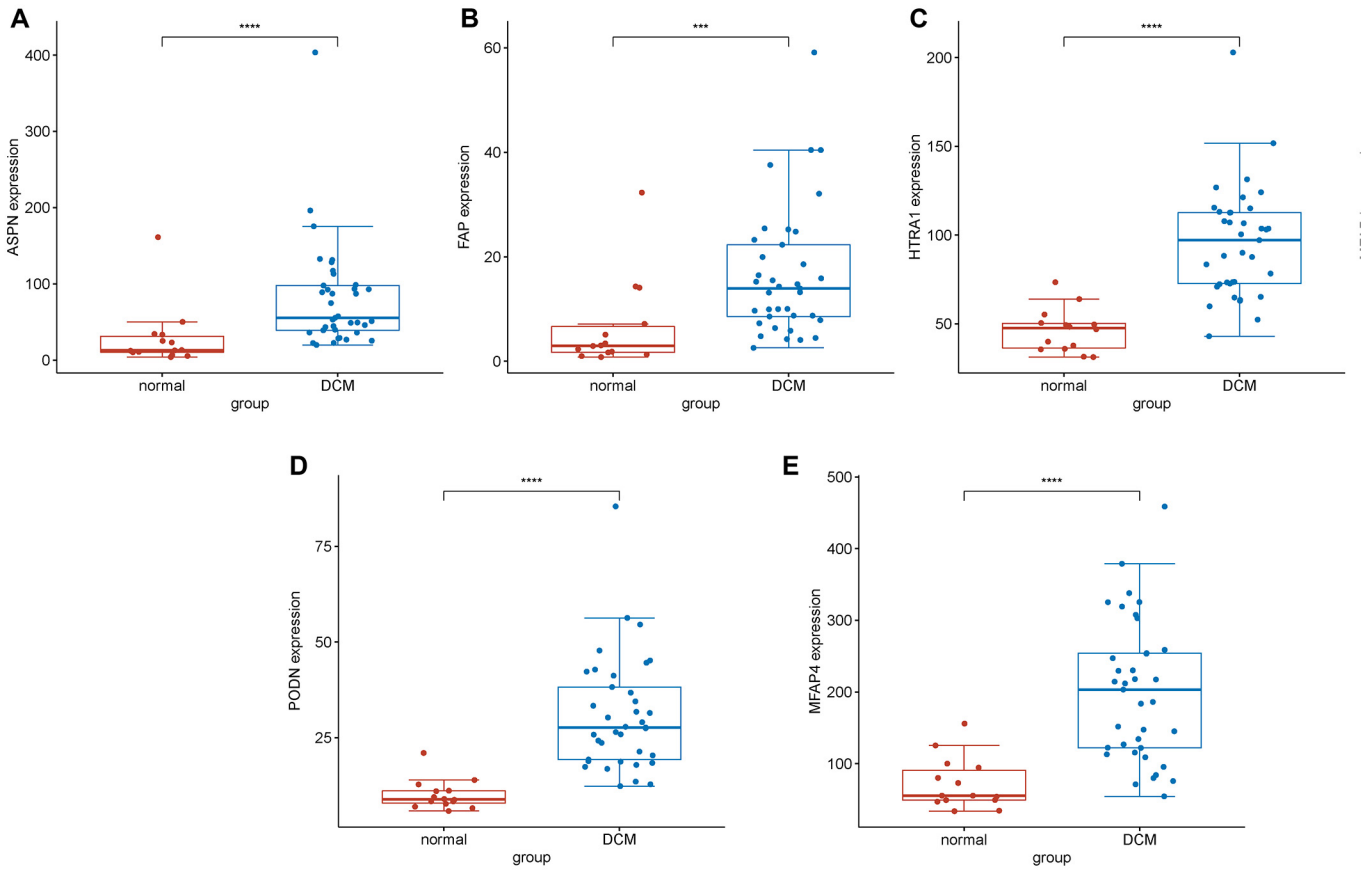
Differential expression analysis of experimentally validated genes in the test group. Expression levels of genes **(A)** ASPN, **(B)** FAP, **(C)** HTRA1, **(D)** PODN, and **(E)** MFAP4 in normal and DCM samples. The expression of these genes was found to be significantly upregulated in DCM samples compared to normal samples (^****^represents *p* < 0.0001; ^***^represents *p* < 0.001).

**Figure 7:**
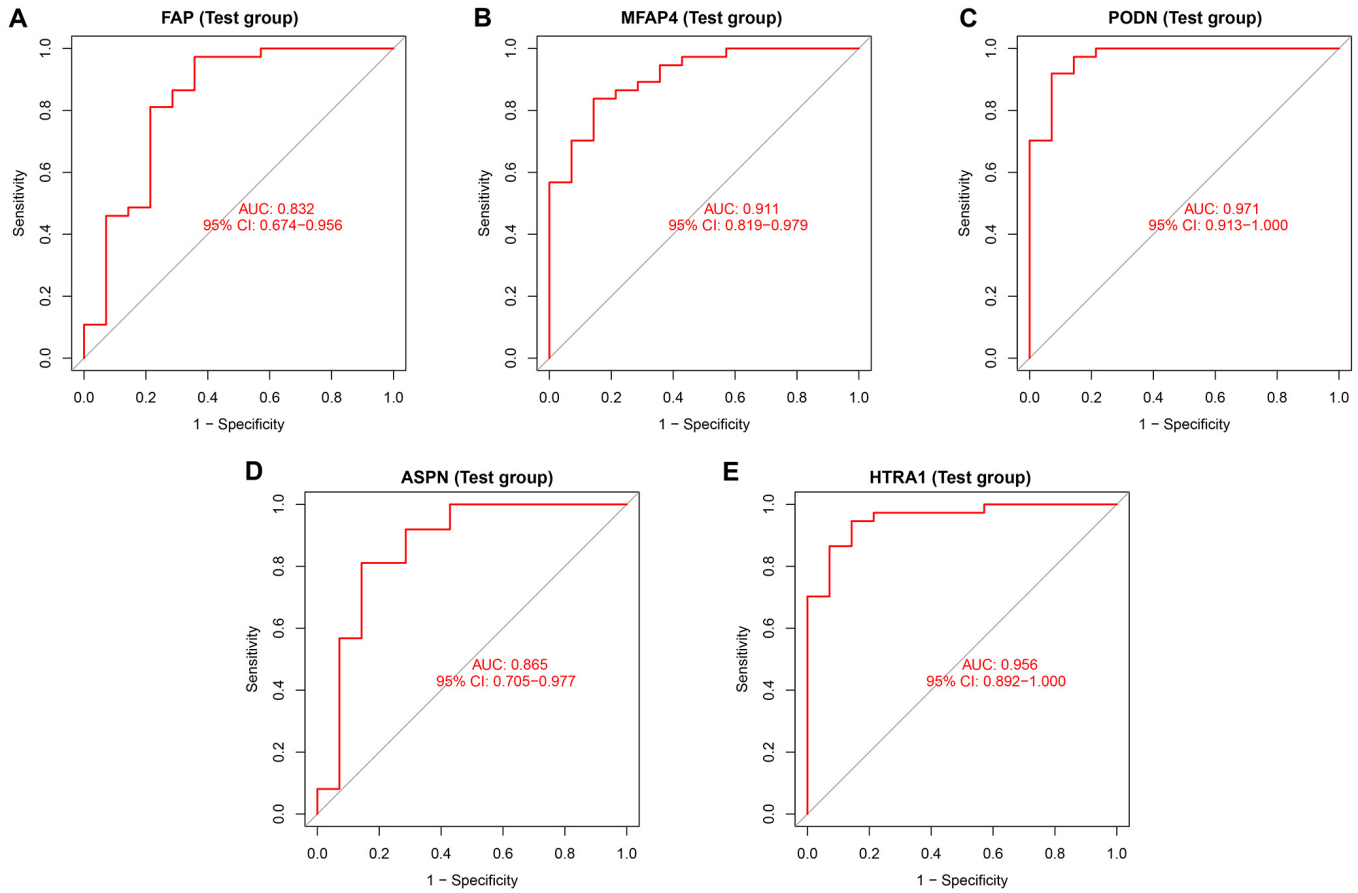
Diagnostic efficiency assessment of validated hub genes in the test group. ROC curve analysis of **(A)** FAP, **(B)** MFAP4, **(C)** PODN, **(D)** ASPN, and **(E)** HTRA1 in the test group.

In both the combined and validation datasets, the expression level of five hub genes (ASPN, MFAP4, PODN, HTRA1, and FAP) was significantly higher in DCM patients compared to healthy individuals (Supplementary Figure S5; Figure 6). In addition, ROC curve results in the train group revealed that these genes exhibited a better diagnostic ability to differentiate DCM from healthy individuals (Supplementary Figure S6). Moreover, the diagnostic efficiency of these genes was validated in the GSE116250, and the AUC of each gene was found to be greater than 0.7 in both the combined and validation datasets (Figure 7).

### 3.7. Immune cell infiltration analysis

**Figure 8:**
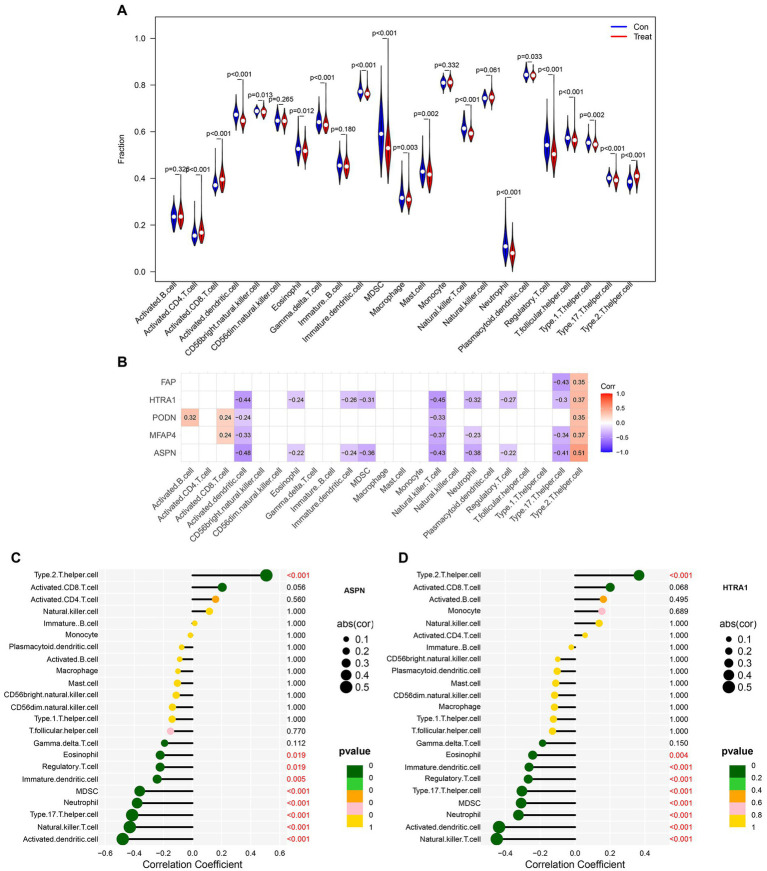
Analysis of immune cell infiltration in patients with DCM. **(A)** Differences in immune microenvironment between healthy and DCM samples. **(B)** Heatmap depicting the correlation between five hub genes and 23 immune cells. Correlation between **(C)** ASPN, **(D)** HTRA1, and infiltrating immune cells in DCM and healthy samples.

The infiltration of immune cells in the myocardium can have detrimental effects on cardiac function (27). Therefore, we sought to determine the infiltration status of immune cells in DCM and normal samples, as well as identified the relationship between the infiltration status of immune cells and the expression level of genes. Between normal and DCM samples, we observed abnormal regulatory levels exhibited by gamma delta T cells, activated CD8 T cells, activated dendritic cells, CD56 bright natural killer cells, eosinophils, activated CD4 T cells, immature dendritic cells, MDSC, macrophages, mast cells, natural killer T cells, neutrophils, plasmacytoid dendritic cells, regulatory T cells, T follicular helper cells, type 1 T helper cells, type 17 T helper cells, and type 2 T helper cells (Figure 8A). Additionally, the association of 23 different types of immune cells was evaluated. The findings showed that the majority of immune cells had a strong synergistic effect, with MDSC and activated dendritic cells having the strongest synergistic effect (Supplementary Figure S7). Furthermore, ASPN and HTRA1 showed a higher correlation with most of the immune cells, as revealed by the relationship between validated hub genes and immune cells (Figure 8B). Lollipop diagrams highlighted the in-depth association between ASPN and immune cells as well as HTRA1 and immune cells (Figures 8C,D). Overall, our results indicate that the five validated hub genes (ASPN, MFAP4, PODN, HTRA1, and FAP) may contribute to the progression of DCM by regulating multiple immune cells.

## 4. Discussion

Dilated cardiomyopathy (DCM) is the leading indication for heart transplantation globally and a major cause of sudden cardiac mortality and heart failure (28). However, the mechanism underlying DCM is not fully understood. DCM may be attributed to genetic or non-genetic factors and the combined effect of genetic susceptibility and environmental factors (29). Therefore, understanding the changes occurring at the genetic level in DCM is essential for developing innovative therapeutic strategies against this disease. We combined two persistent DCM datasets (GSE57338, GSE120895) and employed two distinct techniques to find key genes in our research (WGCNA and LIMMA methods). We identified 322 DEGs between DCM patients and healthy individuals using the LIMMA method, including 164 significantly upregulated and 158 significantly downregulated genes (Supplementary Figure S1). We also discovered six gene modules in the WGCNA, with the blue module (971 genes) having the strongest association with DCM (Figure 1). Thereafter, common genes (CGs) were obtained by intersecting the genes in the most important module, DEGs in the LIMMA method, and the genes of interest. The 42 CGs identified via intersection were then subjected to functional enrichment analysis to investigate their potential regulatory pathways. Based on the GO and KEGG analyses, the pathway was primarily enriched in calcium ion homeostasis, cellular calcium ion homeostasis, positive regulation of cytosolic calcium ion concentration, calcium ion binding, and calcium signaling pathway (Figure 2).

Dilated cardiomyopathy is known to be associated with impaired myocardial function (1), and alterations in systolic proteins and excitation-contraction coupling function are some of the contributing factors (30). Calcium plays an important role in various biological processes, such as synapse formation, vesicle release, and muscular contraction (31). Additionally, Ca^2+^ homeostasis is essential for optimal cardiac excitation-contraction coupling (32). Several studies reported that dysregulated calcium homeostasis is a major contributor to the pathogenesis of DCM (33–36). The maintenance of Ca^2+^ homeostasis is a highly integrated process comprising numerous hormonally controlled feedback loops and a complex system of Ca^2+^-transporters, channels, exchangers, binding/buffering proteins, and pumps. Disruption in any of these components may dysregulate calcium homeostasis (37, 38). Interestingly, our study discovered that numerous genes were enriched in calcium-related pathways, including calcium homeostasis-related pathways, calcium binding, regulation of cytosolic calcium concentration, etc. Additionally, it confirms the critical role that calcium homeostasis plays in the pathogenesis of DCM.

A PPI network was created using CGs to better understand the biological properties of proteins. We identified the top eight hub genes (LTBP2, ASPN, ISLR, MFAP4, FNDC1, PODN, HTRA1, and FAP) using three different algorithms in CytoHubba (Supplementary Figure S3), and RT-qPCR was used to further analyze these hub genes in a DOX-induced DCM animal model (Figure 3; Supplementary Figure S4). In line with the findings from the combined and validation datasets, we discovered that the expression levels of PODN, HTRA1, ASPN, MFAP4, and FAP were considerably higher in DOX animals than in the control group (*p* < 0.05), suggesting that these genes may be involved in the development of DCM. PODN, a member of the small leucine-rich repeat proteoglycans (SLRPs), was recently discovered in the kidney. Subsequently, using reverse transcription-polymerase chain reaction, PODN mRNA was also discovered in additional tissues, such as cardiac and vascular smooth muscle cells, suggesting that PODN may have a potential growth-regulating role in cardiovascular tissues (39). Hutter et al. reported a robust and preferential expression of PODN in the arteries of wild-type mice subjected to injury. Podocan-deficient animals demonstrated accelerated arterial lesion progression in response to injury as compared to wild-type littermates (40). On the other hand, PODN overexpression significantly reduced SMC migration and proliferation in human SMCs by inhibiting the Wnt-TCF (T-cell factor) signaling pathway (40). HTRA1 is a secreted serine protease that is widely expressed and abundant in vascular smooth muscle cells (41). Additionally, HTRA1 is considered to be a pro-fibrotic gene associated with fibrosis (42). HTRA1 not only degrades matrix components and impairs elastogenesis, which results in elastic fiber breakage (43, 44), but also modulates the activation of the TGF-β1 signaling pathway (45, 46). TGF-1, the most potent pro-fibrotic cytokine to date, is one of the key mediators of fibroblast activation and fibrosis in the diseased heart (47). However, it is unclear whether HTRA1 mediates the involvement of TGF-β1 in myocardial fibrosis and myocardial remodeling in DCM. Another hub gene (ASPN), a member of the SLRP family, plays a significant role in tissue injury and regeneration. According to Liu et al. (48), ASPN is the most highly expressed gene in keloids. Overexpression of ASPN inhibits fibroblast activity and differentiation into mature myofibroblasts, which quickly alter the extracellular matrix, thereby resulting in keloid formation and invasion (48). Proteomic research by Manuel Mayr et al. revealed a role for ASPN in cardiac remodeling and validated their finding in patients with ischemic cardiomyopathy. Huang et al. (49) discovered that ASPN mimetic peptides prevented aortic constriction-induced cardiac fibrosis and protected normal cardiac function in mice. Downregulation of ASPN can inhibit abnormal myofibroblast development and protofibroblast gene expression *in vitro*, thereby blocking or reversing the progression of pulmonary fibrosis (50). Another hub gene linked to fibrosis, MFAP4, is a 36 kDa secreted extracellular matrix glycoprotein that belongs to the fibrinogen-related protein superfamily and is involved in the production of elastic fibers (51). According to Dorn et al. (52), the knockdown of MFAP4 resulted in increased ventricular hypertrophy and worsened cardiac function in response to chronic pressure overload. In another study by Zhang et al. (53), plasma and atrial MFAP4 protein levels were increased in atrial fibrosis rats and exhibited a positive correlation with the severity of the condition. MFAP4 deficiency mitigates myocardial fibrosis and ventricular arrhythmias induced by aortic constriction or isoproterenol in mice but does not significantly affect the hypertrophy response (54). FAP is expressed in tissues by activated fibroblasts, and is involved in the healing and remodeling of heart wounds (55). In resting fibroblasts, FAP is expressed at low levels but shows a steep increase in cases of myocardial infarction (56, 57). In rats administered angiotensin II and phenylephrine, ablation of FAP-positive cells lowered cardiac fibrosis and restored systolic function (58). Additionally, FAP has emerged as an intriguing molecular target for diagnosing and treating numerous disorders. Using FAPI-positron emission tomography-computed tomography images, Heckmann et al. (59) examined the activity of FAP in human hearts. In this study, an association between increased FAP signal intensities, cardiovascular risk factors, and metabolic disease was reported (59). Myocardial fibrosis and cardiac remodeling are known as the pathological hallmarks of DCM (1, 60, 61), and the association of hub genes (MFAP4, ASPN, and FAP) with fibrosis and cardiac remodeling has been identified in prior research. Notably, our study discovered that these genes were more highly expressed in the DCM group than in the control group, both in microarray data and the DCM model (Figures 3, 6; Supplementary Figure S5). We, therefore, hypothesized that these genes may contribute to the incidence and progression of DCM by mediating myocardial fibrosis and cardiac remodeling in DCM-affected hearts.

Moreover, we developed a risk prediction model and a neural network model based on five validated hub genes (ASPN, MFAP4, PODN, HTRA1, and FAP). Both models demonstrated good sensitivity and specificity in the train as well as test groups (Figures 4, 5). Our study, however, aims to complement current diagnostic and treatment approaches rather than fully replace them (Figure 7; Supplementary Figure S6). DCM develops slowly, and initially, patients may not have any symptoms. However, once symptoms start appearing, irreparable heart abnormalities have already occurred (62). The applicability of the current diagnostic criteria and procedures is uncertain for DCM patients, particularly in their early stages. The diagnostic and risk assessment models derived from our study enable the possibility of DCM to be detected by timely cardiac biopsy. Thus, our approach holds definite clinical importance. However, based on the current findings, the accuracy of the model warrants further research.

Our study has certain limitations. Bioinformatics is a useful tool for exploring the relationship between genomes and diseases; however, the datasets might have additional limitations and deviations. Moreover, bioinformatics analysis merely serves to provide suggestions and ideas for future studies. The results of bioinformatics studies may vary slightly from the results of the actual experiment. Other factors like sample size and animal model may also impact results. We cannot rule out the possibility that additional genes and pathways excluded from the analyses contribute to the development of DCM. Also, regulators like non-coding RNA or microRNA may influence DCM pathophysiology by regulating the expression of certain genes or underlying molecular mechanisms. Moreover, our study’s primary objective was to develop a risk assessment and diagnostic model for DCM to help with early detection and treatment; we did not look into the specific mechanisms involved in these genes in DCM. Further studies should therefore be conducted on the specific pathways implicated in these genes and the prognostic impact of altered expression of these genes in animal models of DCM. In addition, when we combined different data sets to find DEGs, baseline data were not taken into account, which may have resulted in the loss of some biological information. We could not perform experiments on human samples to further support our findings as it is challenging to obtain human cardiac tissues.

## 5. Conclusion

In summary, our study used LIMMA, WGCNA, and other bioinformatics methods to identify 42 significant CGs between DCM and normal samples, which exhibited the strongest association with DCM. The PPI network was created using these CGs, from which the five most prominent key genes were identified after experimental validation. Furthermore, a DCM risk prediction model and neural network model were developed using these genes, and the diagnostic efficiency of each gene was evaluated in both these models. On being validated, these models and genes demonstrated high accuracy and sensitivity, as well as the ability to assess patients’ risk of developing DCM, thereby facilitating early intervention and therapy. The current findings may reveal an underlying association between DCM and these key genes, which could serve as biomarkers for diagnosing and treating DCM.

## Data availability statement

The datasets presented in this study can be found in online repositories. The names of the repository/repositories and accession number(s) can be found in the article/Supplementary material.

## Ethics statement

The animal study was reviewed and approved by the Institutional Ethics Committee of Chongqing Medical University.

## Author contributions

JC and CY conceived and designed the study. WY and LL performed data analysis as well as wrote the manuscript, and performed the experiments and collected the data. XT and XL corrected the R code. All authors contributed to the article and approved the submitted version.

## Footnotes

^1^ https://www.ncbi.nlm.nih.gov/geo/
