# Supplementary material for: Development and validation of risk prediction and neural network models for dilated cardiomyopathy based on WGCNA
Source: Front Med (Lausanne). 2023 Oct 5;10:1239056. doi: 10.3389/fmed.2023.1239056 (PMC10585101; doi:10.3389/fmed.2023.1239056)
Supplement: Supplementary file 1 [file Data_Sheet_1.docx]

Supplementary Material

# Supplementary Table

# Table S1. The gene expression profile data characteristics

| **Dataset** | **DCM** | **Normal** | **Tissue** | **Platform** |
| --- | --- | --- | --- | --- |
|  |  |  |  |  |
| GSE57338 | 82 | 136 | left ventricle | GPL11532 Affymetrix Human Gene 1.1 ST Array |
| GSE120895 | 47 | 8 | Myocardium | GPL570 Affymetrix Human Genome U133 Plus 2.0 Array |
| GSE116250 | 37 | 14 | left ventricle | GPL16791 Illumina HiSeq 2500 (Homo sapiens) |

# Supplementary Figures


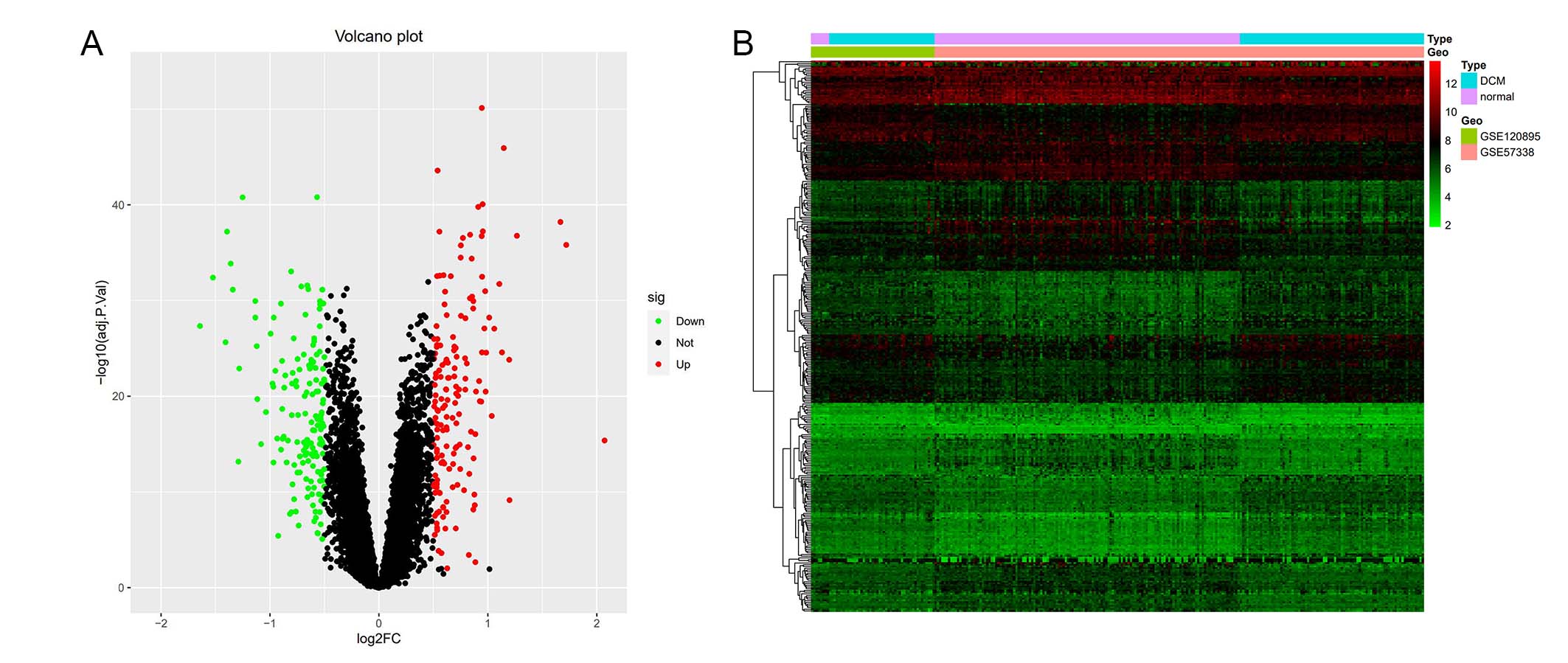


**Figure S1.** The heat map and Volcano plot illustrate the DEGs identified from the integrated dataset. **(A)** Upregulated DEGs are represented by red plot points, while downregulated DEGs are shown by blue plot points. **(B)** Each row of the heat map corresponds to one DEG, and each column represents one sample, either normal or DCM. Upregulated and downregulated DEGs are depicted by red and blue colors, respectively. Abbreviations: DEG, differentially expressed gene; DCM, dilated cardiomyopathy.


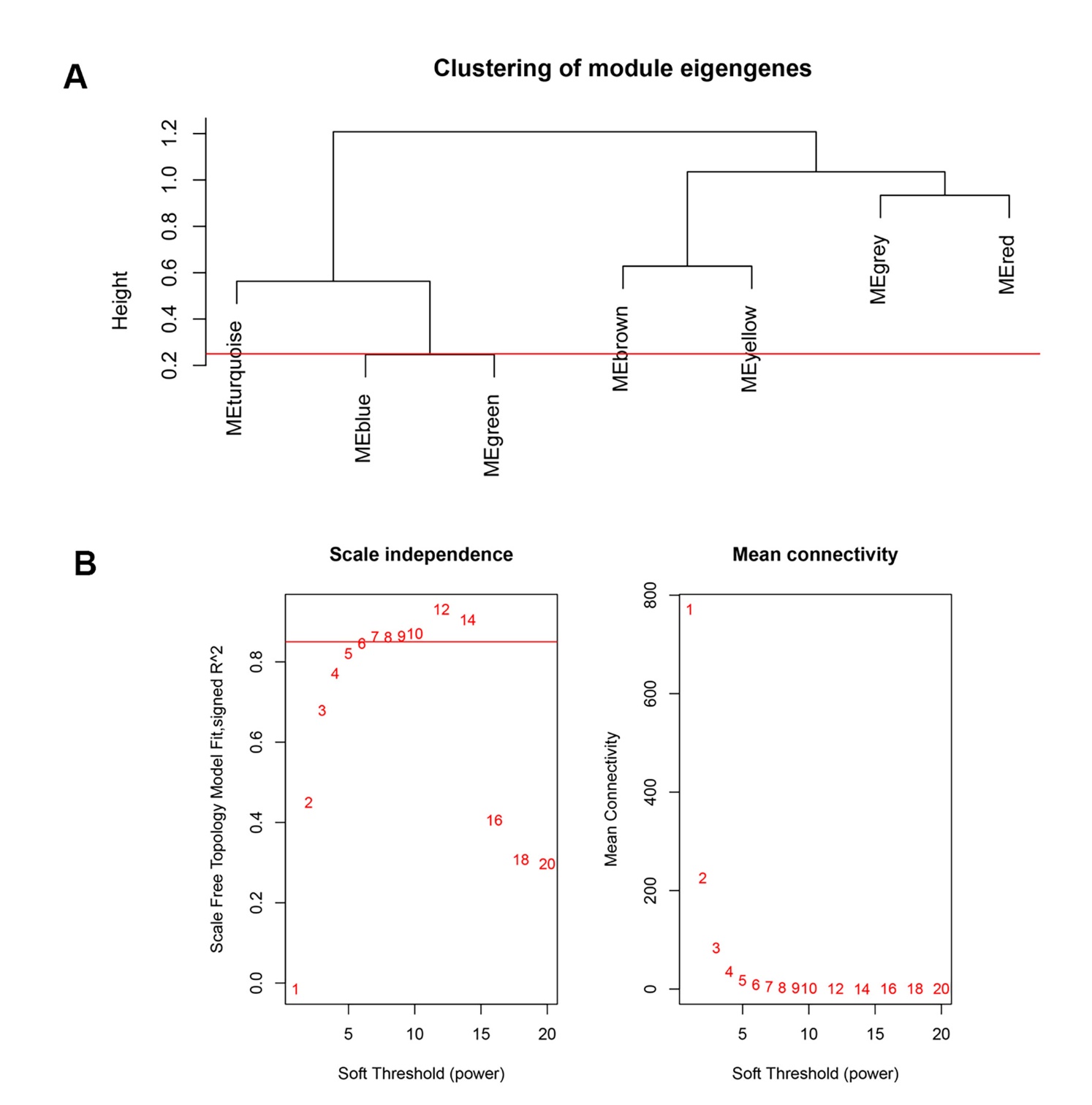


**Figure S2.** Merging of similar modules and screening of optimal power values. **(A)** Consolidation of similar modules. The red line represents the dissimilarity degree with a value of 0.25. Here, we merge the modules that have less than a 25% dissimilarity (blue module and green module). **(B)** Selection of optimal soft thresholds. The red line represents R^2 =^0.85. Here, we choose the optimal power value β= 7.


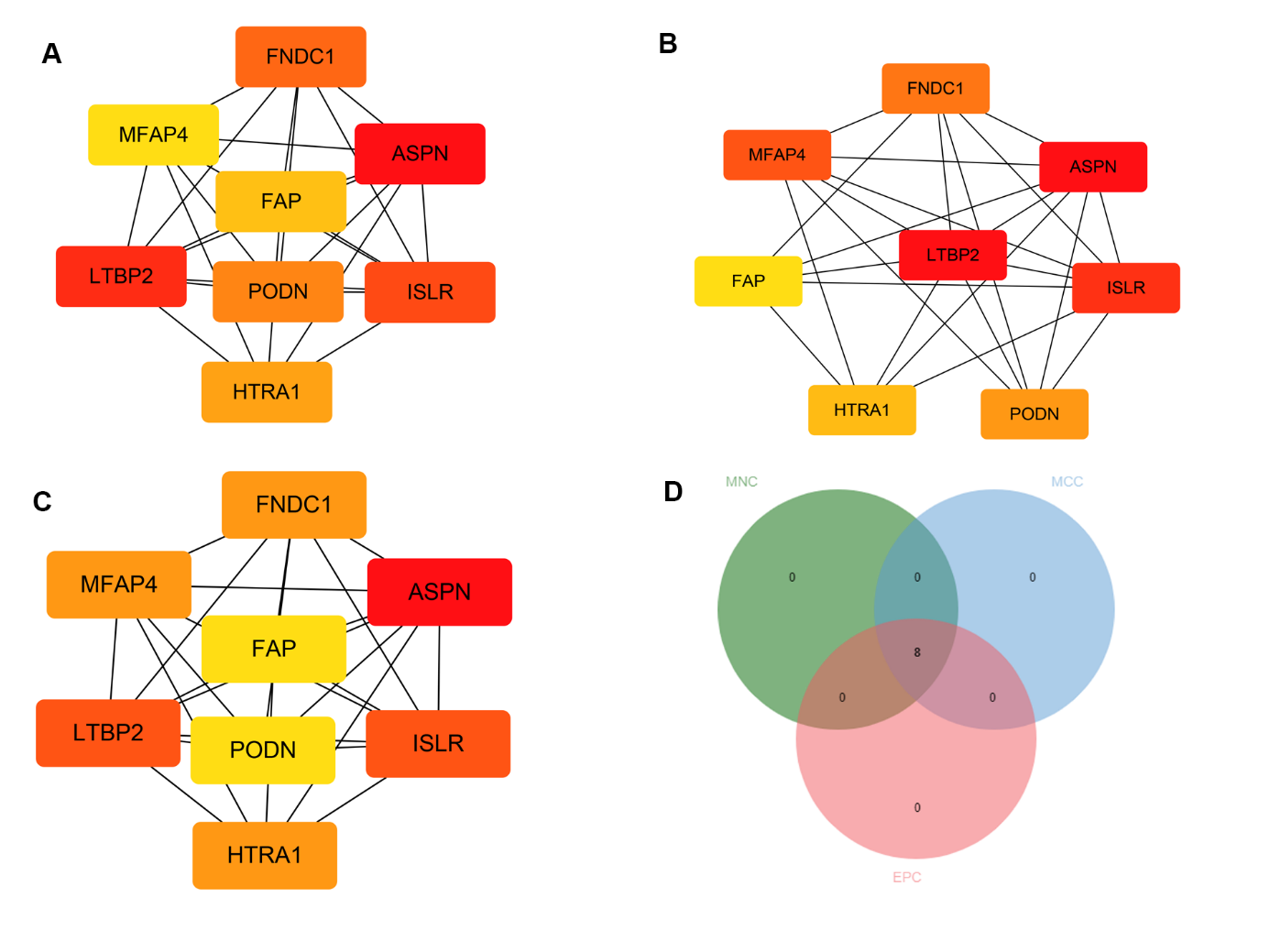


**Figure S3.** Analysis of interaction network using different methods in CytoHubba. **(A)** Edge Percolated Component (EPC). **(B)** Maximum Group centrality (MCC). **(C)** Maximum Neighborhood Component (MNC). **(D)** The Venn diagram depicts the intersection of the hub genes.


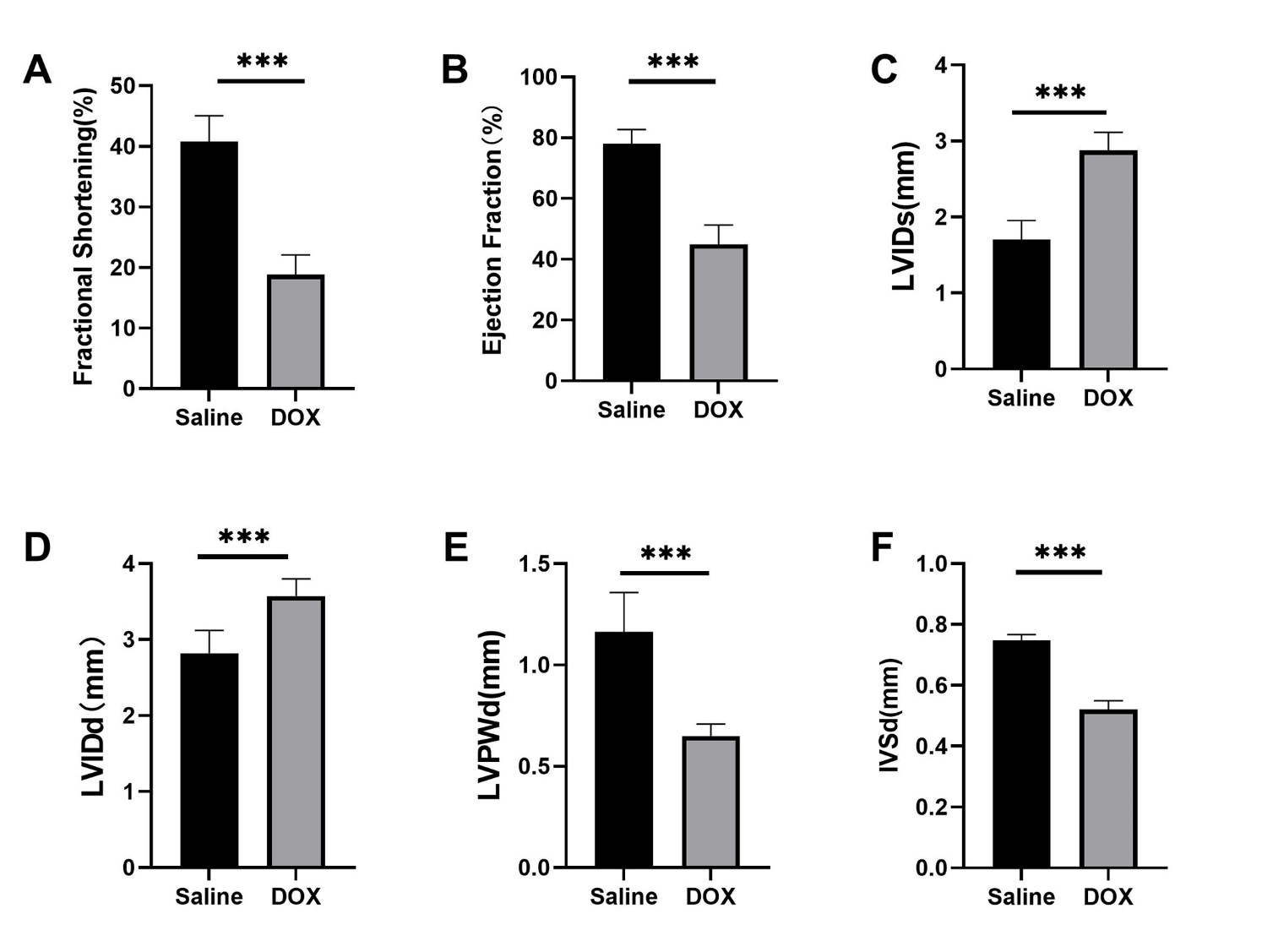


**Figure S4**. Ultrasound examination of DCM mouse (DCM group, n = 6; control group, n = 6). **(A)** FS, **(B)** EF, **(C)** LVIDs, **(D)** LVIDd, **(E)** LVPWd, and **(F)** IVSd in DCM or normal mice. Data are presented as the mean ±standard deviation. FS, fractional shortening; EF, Ejection fraction; LVIDs/d, left ventricular internal diameter end systole/diastole; LVPWd, left ventricular posterior wall end diastole; IVSd, interventricular septal end diastole; DOX, doxorubicin. (***represents: p < 0.001; **represents p < 0.01; *represents p < 0.05).


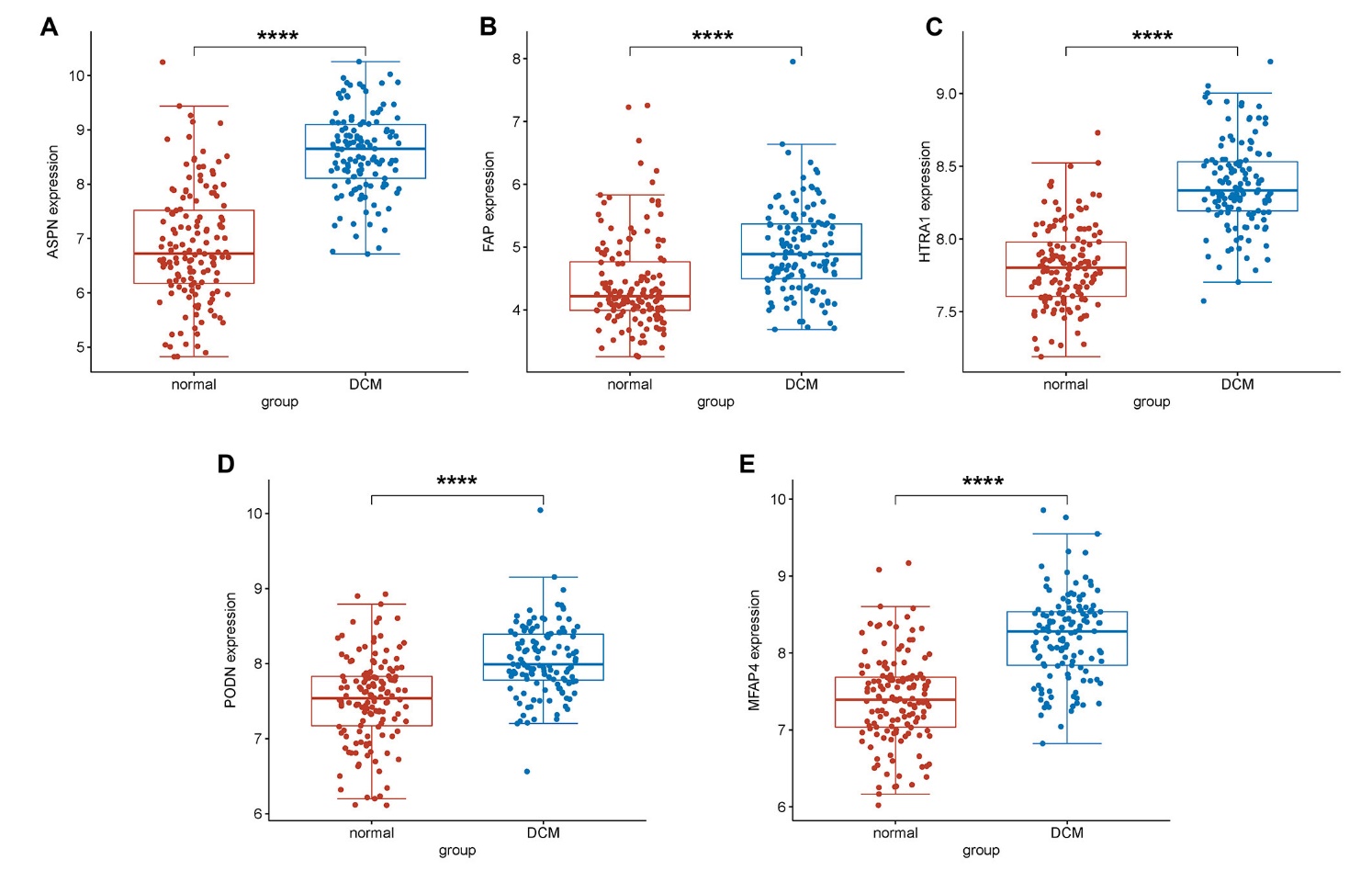


**Figure S5.** Analysis of differential expression of experimentally validated genes in the train group. Gene expression levels of **(A)** ASPN, **(B)** FAP, **(C)** HTRA1, **(D)** PODN, and **(E)** MFAP4 between normal and DCM samples. The expression of these genes was significantly upregulated in DCM samples compared to normal samples. (****represents: p < 0.0001).


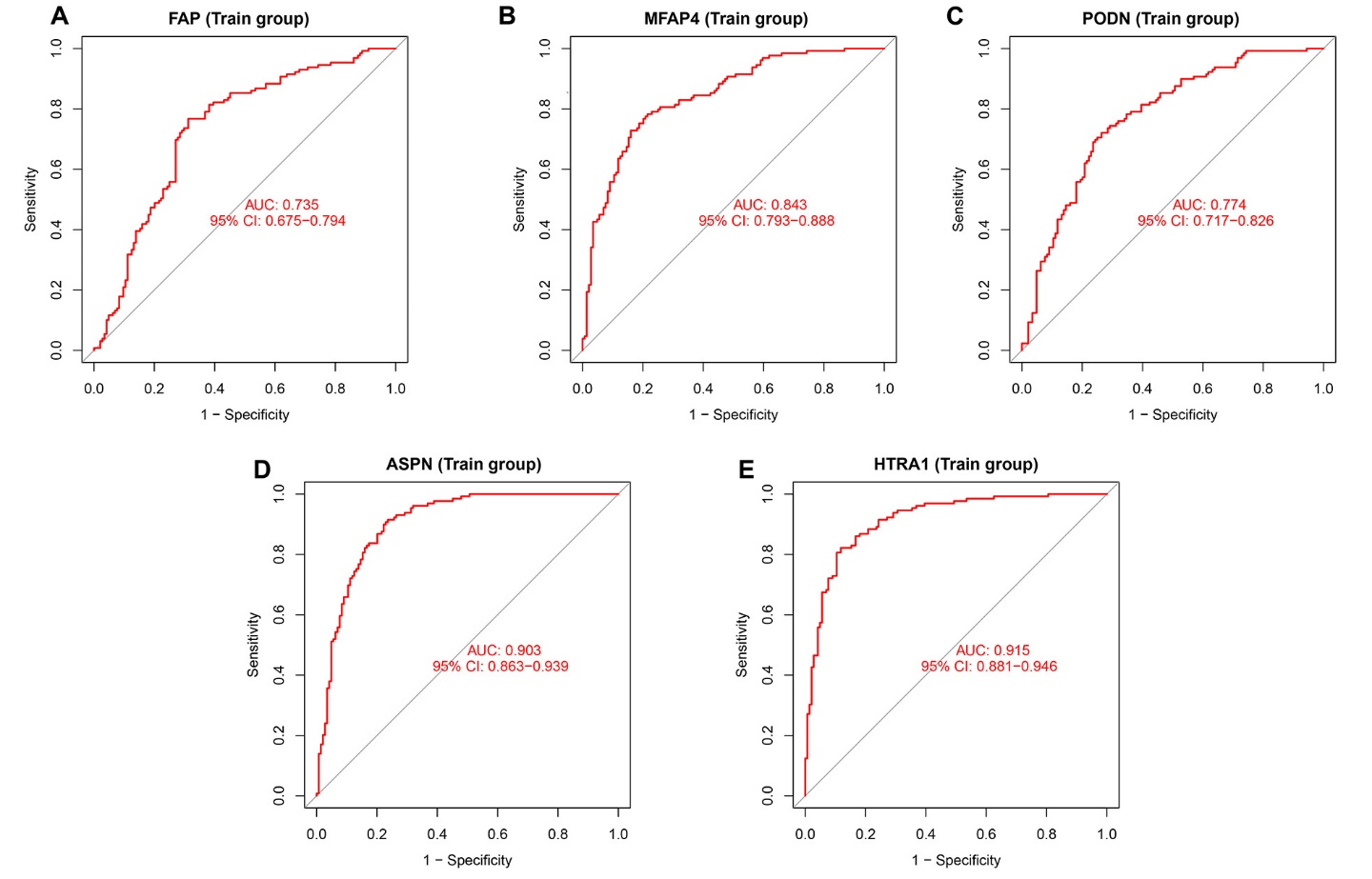


**Figure S6**. Diagnostic efficiency assessment of validated hub genes in the train group. ROC curve analysis of **(A)** FAP, **(B)** MFAP4, **(C)** PODN, **(D)** ASPN, and **(E)** HTRA1 in the merged dataset.


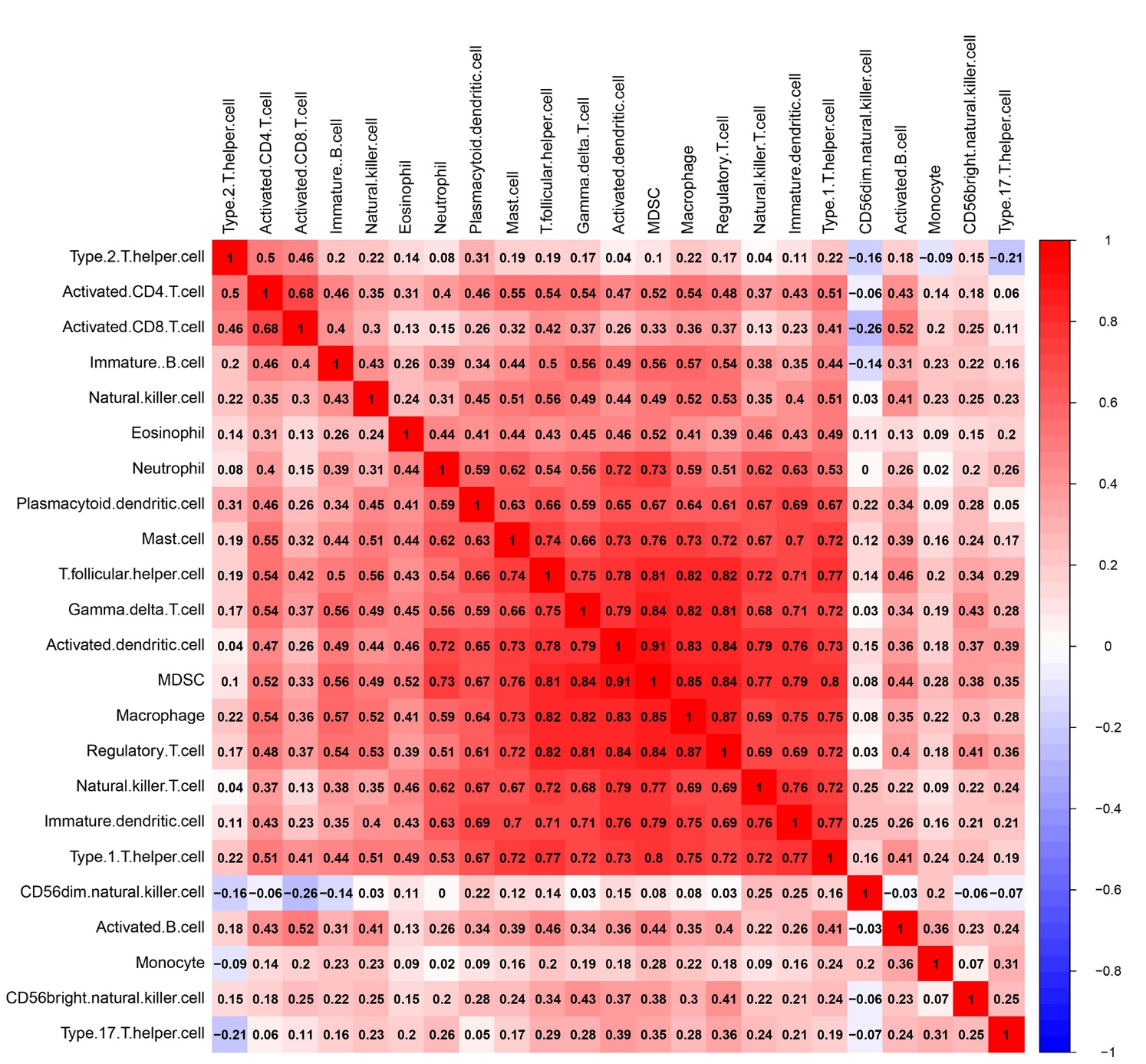


**Figure S7.** Heat map of the correlation between immune cells. As shown in the figure, MDSC had the highest correlation with activated dendritic cells (correlation coefficient =0.91).
